# A Phase II Trial of a Personalized, Dose-Intense Administration Schedule of ^177^Lutetium-DOTATATE in Children With Primary Refractory or Relapsed High-Risk Neuroblastoma–LuDO-N

**DOI:** 10.3389/fped.2022.836230

**Published:** 2022-03-10

**Authors:** Fredrik Sundquist, Kleopatra Georgantzi, Kirsten Brunsvig Jarvis, Jesper Brok, Minna Koskenvuo, Jelena Rascon, Max van Noesel, Per Grybäck, Joachim Nilsson, Arthur Braat, Mikael Sundin, Sandra Wessman, Nikolas Herold, Lars Hjorth, Per Kogner, Dan Granberg, Mark Gaze, Jakob Stenman

**Affiliations:** ^1^Department of Women's and Children's Health, Karolinska Institutet, Stockholm, Sweden; ^2^Pediatric Oncology, Astrid Lindgren Children's Hospital, Karolinska University Hospital, Stockholm, Sweden; ^3^Department of Paediatric Haematology and Oncology, Oslo University Hospital, Rikshospitalet, Oslo, Norway; ^4^Department of Paediatrics and Adolescent Medicine, Rigshospitalet, Copenhagen, Denmark; ^5^Helsinki University Central Hospital, University of Helsinki, Helsinki, Finland; ^6^Center for Pediatric Oncology and Hematology, Vilnius University Hospital Santaros Klinikos, Vilnius, Lithuania; ^7^Solid Tumor Department, Princess Máxima Center for Pediatric Oncology, Utrecht, Netherlands; ^8^Department of Medical Radiation Physics and Nuclear Medicine, Karolinska University Hospital, Stockholm, Sweden; ^9^Department of Nuclear Medicine, Princess Máxima Center for Pediatric Oncology, Utrecht, Netherlands; ^10^Division of Pediatrics, Department of Pediatric Hematology, Immunology and HCT, Department of Clinical Science, Intervention and Technology (CLINTEC), Karolinska University Hospital, Karolinska Institutet, Stockholm, Sweden; ^11^Department of Pathology, Department of Oncology-Pathology, Karolinska University Hospital, Karolinska Institutet, Stockholm, Sweden; ^12^Department of Clinical Sciences Lund, Paediatrics, Lund University, Skane University Hospital, Lund, Sweden; ^13^Department of Breast, Endocrine Tumors and Sarcomas, Department of Molecular Medicine and Surgery, Karolinska University Hospital, Karolinska Institutet, Stockholm, Sweden; ^14^Department of Oncology, University College London Hospitals NHS Foundation Trust, London, United Kingdom; ^15^Department of Pediatric Surgery, Karolinska University Hospital, Stockholm, Sweden

**Keywords:** neuroblastoma, relapse, refractory, ^177^Lu-DOTATATE, PRRT, radiopharmaceutical, therapy, high-risk

## Abstract

**Background:**

Half the children with high-risk neuroblastoma die with widespread metastases. Molecular radiotherapy is an attractive systemic treatment for this relatively radiosensitive tumor. ^131^I-mIBG is the most widely used form in current use, but is not universally effective. Clinical trials of ^177^Lutetium DOTATATE have so far had disappointing results, possibly because the administered activity was too low, and the courses were spread over too long a period of time, for a rapidly proliferating tumor. We have devised an alternative administration schedule to overcome these limitations. This involves two high-activity administrations of single agent ^177^Lu-DOTATATE given 2 weeks apart, prescribed as a personalized whole body radiation absorbed dose, rather than a fixed administered activity. “A phase II trial of ^177^Lutetium-DOTATATE in children with primary refractory or relapsed high-risk neuroblastoma - LuDO-N” (EudraCT No: 2020-004445-36, ClinicalTrials.gov Identifier: NCT04903899) evaluates this new dosing schedule.

**Methods:**

The LuDO-N trial is a phase II, open label, multi-center, single arm, two stage design clinical trial. Children aged 18 months to 18 years are eligible. The trial is conducted by the Nordic Society for Pediatric Hematology and Oncology (NOPHO) and it has been endorsed by SIOPEN (https://www.siopen.net). The Karolinska University Hospital, is the sponsor of the LuDO-N trial, which is conducted in collaboration with Advanced Accelerator Applications, a Novartis company. All Scandinavian countries, Lithuania and the Netherlands participate in the trial and the UK has voiced an interest in joining in 2022.

**Results:**

The pediatric use of the Investigational Medicinal Product (IMP) ^177^Lu-DOTATATE, as well as non-IMPs SomaKit TOC® (^68^Ga-DOTATOC) and LysaKare® amino acid solution for renal protection, have been approved for pediatric use, within the LuDO-N Trial by the European Medicines Agency (EMA). The trial is currently recruiting. Recruitment is estimated to be finalized within 3–5 years.

**Discussion:**

In this paper we present the protocol of the LuDO-N Trial. The rationale and design of the trial are discussed in relation to other ongoing, or planned trials with similar objectives. Further, we discuss the rapid development of targeted radiopharmaceutical therapy and the future perspectives for developing novel therapies for high-risk neuroblastoma and other pediatric solid tumors.

## Introduction

Neuroblastoma (NBL) is a malignant disease that most commonly occurs in early childhood, and displays a wide heterogeneity regarding biological features, clinical presentation, morbidity, and mortality (1). It derives from neural crest cells, precursors of the sympatico-adrenergic system, manifesting in the adrenal glands and/or along the para-vertebral sympathetic ganglia (1). With an incidence of ~10 cases per million children under 15 years of age, approximately 1 in 7,000 children are affected (2). NBL is risk-stratified in terms of historical outcome in low-, intermediate- or high-risk based on age, stage, histologic classification, grade of tumor differentiation, MYCN amplification and chromosome 11q deletion status, according to The International Neuroblastoma Risk Group (INRG) Classification System (3). At least half of NBL patients are high-risk (HR). Treatment of HR-NBL generally consists of (i) an induction phase with intensive neoadjuvant chemotherapy, (ii) establishment of local control by surgery and radiotherapy, (iii) systemic remission consolidation therapy with high-dose chemotherapy and autologous stem cell reinfusion, and (iv) maintenance therapy with anti-GD2 antibodies and retinoic acid (3–5). In spite of this multimodal treatment, survival has remained at ~50% in HR-NBL. In case of a relapse, primary refractory disease or ultra-high-risk features, the outcome is dismal (1, 3, 4, 6). Historically treatment options for relapsed or refractory disease have focused on further cytotoxic chemotherapy with unsatisfying outcomes, partly due to chemo-resistance and poor bone marrow reserve after prior therapies and bone marrow disease (7). There is no consensus regarding the most effective therapy in this setting and currently two other European trials are focusing on this group of patients: The VERITAS trial comparing the combination of cytotoxic chemotherapy and intravenous targeted ^131^I-mIBG radiotherapy with high-dose cytotoxic chemotherapy; *(VERITAS trial, Institute Gustave Roussy, Paris, France*, *ClinicalTrials.gov* *Identifier: NCT03165292)* and the MINIVAN trial that compares different combinations of anti-PD1 and anti-GD2 therapy and intravenous targeted ^131^I-mIBG radiotherapy, *(MINIVAN trial, University Hospital Southampton NHS Foundation Trust, UK*, *ClinicalTrials.gov* *Identifier: NCT02914405)*. In addition, the BEACON trial comparing the combination of cytotoxic chemotherapy and anti-VEGF therapy with cytotoxic chemotherapy alone; *(BEACON trial, University of Birmingham*, *ClinicalTrials.gov* *Identifier: NCT02308527)* was recently finalized. Also, a multinational trial evaluating the combination of Naxitamab (anti-GD2) and GM-CSF (YmAbs Therapeutics, *ClinicalTrials.gov* *Identifier: NCT03363373)* is currently recruiting in several European countries.

Neuroblastoma is known to be a radiosensitive disease, although if the disease is localized in several different sites external beam radiotherapy is not suitable. Therefore, targeted molecular radiotherapy is a more attractive treatment option in this setting, as it delivers tumor specific radiation and can target multiple sites of disease from the same administration (8–14). Studies have shown that 85–90% of neuroblastomas express the noradrenaline transporter molecule and can be targeted with the catecholamine analog, meta-iodobenzylguanidine (mIBG), labeled with ^123^I for imaging and with the β-emitter ^131^I for therapy. Molecular radiotherapy with ^131^I-mIBG has been used in relapsed and refractory neuroblastoma since the mid 1980's (15). Published data includes results from Phase I, Phase II trials and Pilot Studies with reported response rates ranging from 0 to 60% but there has been a wide variation in the administered radiation doses and methods of response assessment (16–20). The dose limiting toxicity for ^131^I-mIBG is confined to the bone marrow and attempts to increase the response rate to ^131^I-mIBG have included dose escalation and the use of radio-sensitizing agents, for example Topotecan. Both of these approaches require stem cell support to circumvent myelotoxicity (21, 22).

Another alternative target for molecular radiotherapy in neuroblastoma is the somatostatin receptors. Prior studies during the 1990's characterized the expression of somatostatin receptors in neuroblastoma cell lines and *in vivo* scintigraphic tumor imaging suggested putative therapeutic applications. Further studies confirmed that somatostatin analog therapy was effective in neuroblastoma xenografts *in vivo* (23–27). Recent studies based on immunohistochemistry have demonstrated expression of all known somatostatin receptors SSTR 1–5 on primary neuroblastoma (28). More specifically, SSTR 2 is expressed in the majority of neuroblastomas, even in recurrent tumors and in primary refractory disease (29). This can be utilized according to the same principle as ^123^I- and ^131^I-mIBG, by targeting SSTR with somatostatin analogs that can be labeled with ^68^Ga for imaging and with the β-emitter ^177^Lu for therapy. ^177^Lu-DOTATATE is composed of a somatostatin analog (TATE) and a chelator (DOTA) that binds the radio-isotope Lutetium-177. TATE binds to somatostatin receptors 1–5 (30), the binding between TATE and SSTR-2 having the highest affinity. The cytotoxic effect of Lutetium is primarily caused by emission of β-radiation. The effective range of the β-radiation is about 0.67 mm, causing a local effect on approximately 10^6^ cells. About 10% of the radiation energy emitted is γ radiation that can be detected by SPECT imaging and utilized for dosimetry by determination of radiation dosage to the tumor target sites and to organs at risk. PET/CT, using the tracer ^68^Ga-DOTATATE or DOTATOC, another somatostatin analog that binds to SSTR receptors, can be used for identifying patients eligible for treatment with ^177^Lu-DOTATATE and this method can also be used for evaluating the response to treatment (31). ^177^Lu-DOTATATE therapy is thus, theoretically analogous to the well-established mIBG-therapy, but it utilizes a different targeting molecule and also a different radio-isotope for delivering beta radiation specifically to the tumor lesions.

^177^Lu-DOTATATE targeting the SSTRs has been shown to be effective in the treatment of neuroendocrine tumors in adults. This type of tumor-specific radiation therapy, called peptide receptor radionuclide therapy (PRRT), has recently been established as second-line treatment for grade 1 and 2 midgut neuroendocrine tumors (NET), that progress after first-line treatment with octreotide (32). ^177^Lu-DOTATATE has been well-tolerated in adult patients with a low incidence of hematological and renal toxicity, which are the main reported side effects. Renal irradiation is compounded by proximal tubular reabsorption of the radiolabeled somatostatin analog and co-administration of amino acid solution has been shown to reduce the risk of renal damage, as has limiting the estimated cumulative radiation dose to the kidneys to 23 Gy or less. The administration of cationic amino acids saturates the renal tubular uptake of proteins/peptides and amino acids, thereby reducing the exposure to the radionuclide (33–35). Severe delayed complications such as secondary malignancies and renal insufficiency has been reported in adult cohorts, but in general, severe side effects are rare. Brabander et al. (36) found that 1.5% of patients developed myelodysplastic syndrome and 0.7% developed acute leukemia during a median follow-up of 5 years after Lu-DOTATATE treatment in a cohort of 610 patients. No therapy-related long-term renal or hepatic failure occurred. A study by Garske-Roman et al. (37) found similar frequencies in a cohort of 200 patients and a median follow-up of 31 months. In this cohort 4% of patients developed grade 2 and 0.5% grade 4 renal toxicity (30, 36, 37). In a recent report from the NETTER-1 Trial, 2% of 111 patients developed myelodysplastic syndrome, whereas no cases of acute myeloid leukemia were reported. One patient developed diffuse large B-cell lymphoma during long-term follow-up, deemed not related to the ^177^Lu-DOTATATE treatment. The long-term renal function was similar in treatment and control groups, but the number of evaluable patients was small (38).

Pilot studies on pediatric patients, performed by Gains et al. at the University College London Hospitals NHS Trust, UK (UCLH) and by Kong et al. the Royal Children's Hospital, Melbourne, Australia (RCH) have shown that ^68^Ga-DOTATOC or DOTATATE PET/CT is a feasible method to identify patients with neuroblastoma that can benefit PRRT with ^177^Lu-DOTATATE. Both of these studies reported promising results from treating children with refractory or relapsed neuroblastoma with ^177^Lu-DOTATATE, some of which achieving remission that lasted for several years (39, 40). A phase IIa LuDO trial at UCLH, did not, however, show an objective response among 14 evaluable patients (41). In this study, dose limiting toxicity was observed only in one patient and the investigators speculate that this may have been due to concurrent use of myelosuppressive antibiotics, rather than an actual side effect of the trial treatment. The measured renal radiation dose was lower than the objective in all cases and the median value was only about 70% of the 23 Gy objective. The main reason for this was the rapid disease progression in many of the study subjects, indicating that with an intensified dosing schedule, the administered activity could have been substantially increased in most patients. Despite the negative result, the investigators conclude that ^177^Lu-DOTATATE may have value as a treatment for neuroblastoma and propose further clinical trials to be conducted (29). The design of the LuDO-N Trial, described in this paper, builds on the experience of the recent LuDO-Trial.

## Methods and Analysis

### Study Design and Setting

The LUDO-N Trial (EudraCT number: 2020-004445-36, ClinicalTrials.gov Identifier: NCT04903899) is a prospective, non-blinded, open-label, single arm two stage, multicenter phase II trial for pediatric patients between 18 months and 18 years old with relapsed or refractory high-risk neuroblastoma. Patients are recruited from the Nordic Society of Pediatric Hematology and Oncology (NOPHO) catchment area, including the Nordic countries and Lithuania, and from the Netherlands with a combined population of roughly 50 million. The LuDO-N Trial is sponsored by the Karolinska University Hospital (Stockholm, Sweden). Study centers have been established in each of the participating countries and the ^177^Lu-DOTATATE treatment will be given at the Karolinska University Hospital, Stockholm Sweden, the Princess Máxima Center, Utrecht, The Netherland and possibly at a later stage at the University College London Hospitals, London, UK. The trial aims to confirm the dose and assesses the response to ^177^Lu-DOTATATE (provided by Advanced Accelerator Applications, a Novartis company) as a single agent for treatment of relapsed or refractory high-risk NBL in children. The pediatric use of the Investigational Medicinal Product (IMP) ^177^Lu-DOTATATE, as well as non-IMPs SomaKit TOC® (^68^Ga-DOTATOC) and LysaKare® amino acid solution for renal protection, have been approved for the LuDO-N Trial by the European Medicines Agency (EMA). Competent authority and ethical approvals and are being applied for separately in each of the participating countries. Fourteen patients will be recruited in the first stage and provided a response, as defined by the Revised International Neuroblastoma Response Criteria (INRC) 1 month after completion of therapy, is seen in 3 or more of these patients, a further 10 will be enrolled in the second stage (42, 43). Accrual is expected to last for up to 60 months. Follow-up continues until 5 years after the last ^177^Lu-DOTATATE treatment or death, of all included patients, whichever occurs first. At writing, the LuDO-N Trial has opened for recruitment in Sweden and Norway and the first patient has recently received ^177^Lu-DOTATATE treatment within the trial.

### Consent and Screening

Participants are screened and recruited by the National Principal Investigator (PI), or delegate, in each of the participating countries. It is the responsibility of the national PI to give oral and written information about the trial and to obtain written informed consent for each patient to be registered. No trial specific procedure is carried out prior to the consent form being signed by the patient and/or parents or legal guardians. All patients undergo the following assessments as part of screening. Please see Tables 1, 2.

**Table 1:** Screening.

**Screening**	**Method**	**Timeframe**
Imaging	^123^I-MIBG	Within 2 months prior to trial treatment
	^68^Ga DOTATOC PET	Within 2 months prior to registration and within 2 weeks prior to trial treatment
	MRI/CT	Within 2 months prior to trial treatment
Bone marrow	Aspirate and trephines According to INSS guidelines	Within 2 months prior to trial treatment
Kidney function	GFR according to recognized local method eGFR	Within 2 months prior registration Prior to trial treatment
Clinical status	Clinical status, vital sign, weight, and performance status.	Within a week prior to registration and prior to each treatment
Urine catecholamine metabolites	Dopamine, HVA, VMA	Within 2 months prior to trial treatment
Blood tests	Hematology, biochemistry	Within a week prior to trial treatment
Pregnancy test		Within a week prior to Cycle 1 dosing
Peripheral blood stem cells		Available at start of trial treatment

**Table 2:** Inclusion and Exclusion criteria.

**Rule**	**Criterium**	**Definition**
Inclusion	Pathology	• Histologically confirmed diagnosis of neuroblastoma
	Stage	• Relapsed or primary refractory high-risk neuroblastoma: INSS stage 4 disease or INRGSS stage M disease
	Age	• Age >18 months and <18 years
	Life expectancy	• >3 months
	Performance status	• Karnofsky >50% or Lansky >50%
	Prior treatment	• 2 weeks since prior treatment • Recovered from prior hematological toxicity • Recovered from major surgery
	Diagnostic imaging	• Uptake in the primary tumor or metastatic tumor deposits on ^68^Ga-DOTATATE PET/CT exceeding the liver uptake and performed within two months prior to registration • ^123^I-mIBG scintigraphy to be performed within two months prior to registration • CT or MRI of the primary tumor and bulky metastatic sites within 2 months prior to registration
	Biochemistry	Hematology: • Hemoglobin, If Hb is <120 g/L then patient will receive a blood transfusion prior to commencing trial treatment • Absolute neutrophil granulocyte count > 1.0 x 10^9^/L • Absolute platelet count > 100 x 10^9^/L Biochemistry: • Bilirubin within 1.5 x ULN • ALT within 2.5 x ULN • AST within 2.5 x ULN • GGT within 5 x ULN • ALP within 5 x ULN • Glomerular filtration rate >50 mL/min/1.73m^2^ assessed by a recognized method, such as inulin, ^51^Cr-EDTA, ^99m^Tc-DTPA or Iohexol clearance and performed within 2 months prior to registration • Urinary catecholamine metabolites measured within 2 months prior to registration
	Peripheral blood stem cells	• A minimum of 4 x 10^6^ CD34 + cells/kg (optimally 6 x10^6^ CD34+ cells/kg) must be available for each study subject prior to registering
	Consent	• Written informed consent from patient and/or parent(s) or legal guardian(s) in accordance with national regulations, prior to registration or any trial-related screening procedures
Exclusion	Pregnant	• Pregnant or lactating patient
	Condition	• Not fit enough to undergo proposed study treatment, as assessed by national PI.
	Concurrent treatment	• Concurrent treatment with any anti-tumor agents
	Prior treatment	• Treatment with long-acting somatostatin analogs within 30 days prior the administration of ^177^Lu-DOTATATE • Prior treatment with other radiolabeled somatostatin analogs
	Allergy	• Hypersensitivity to any component of the investigational drug ^177^Lu-DOTATATE
	Compliance	• Any psychological, familial, sociological or geographical condition potentially hampering compliance with the study protocol and follow-up schedule

### Radiation Safety

Radiation exposure is governed by Swedish national legislation, as defined in the Swedish Radiation Safety Authority regulations SSMFS 2018:1 and 2018:5, in accordance with the European Council Directive 2013/59/EURATOM. It is recognized that children receiving molecular radiotherapy will require care and support from adults whilst receiving their treatment. Children receiving ^177^Lu-DOTATATE will be radioactive and will also have radioactive bodily products such as urine, saliva and vomit, all of which represent a potential radiation hazard to those adults caring for them during their treatment. Potential Supporting Persons will be informed of the risks of radiation exposure and trained in radiation hygiene and appropriate precautions to keep their personal radiation exposure as low as reasonably achievable – the ALARA principle. Patients will require specific medical interventions during their treatment from doctors, nurses and other health care professionals. These individuals will therefore receive some radiation during these tasks. Health care professionals are governed by annual radiation limits and it is essential, in keeping with the ALARA principle, that they do not receive avoidable radiation. Therefore, Supporting Persons are asked to undertake all normal child-care tasks such as washing, dressing, feeding, entertainment and support during the first 24 h after ^177^Lu-DOTATATE administration.

### Study Interventions

A baseline ^68^Ga-DOTATOC PET/CT is performed within 2 weeks, prior to ^177^Lu-DOTATATE treatment. A total of two doses of ^177^Lu-DOTATATE are administered intravenously 2–4 weeks apart. A weight-based activity of 200 MBq kg^−1^ is used for the first dose. The activity of the second dose is calculated based on whole body activity scans as well as SPECT CT scans to determine the absorbed kidney dose. The aim is to administer ^177^Lu-DOTATATE corresponding to a whole-body dose of 1,2 Gy, with a cumulative whole-body dose of about 2,4 Gy over two courses, and not exceeding a cumulative renal dose of 23 Gy, in order to avoid renal toxicity (41). Please see Table 3. LysaKare® amino acid solution for renal protection is administered as a 4-h infusion of 20 ml/kg, which corresponds to typical fluid resuscitation dose in children. This dose translates to the established adult dose of 1,000 ml in a child weighing 50 kg.

**Table 3:** Administration schedule.

**Day**	**Action**	**Definition**
−14– −1	^68^Ga-DOTATOC PET/CT	Baseline
0	^177^Lu-DOTATATE **dose 1**	Dosage: **200** MBq kg^−1^
+1–7	SPECT/CT	Minimum of 3 images
+1–14	Assessment of clinical status/condition/biochemistry	According to trial protocol, see **Supplementary**
+14–28	^177^Lu-DOTATATE **dose 2**	Dosage: **x** MBq kg^−1^, **x** calculated from SPECT/CT, target maximum dose **≤2,4 Gy to whole-body** (calculated from image data) AND **≤2,4 Gy to whole-body** (calculated from dose rate survey readings) AND **≤23 Gy to either kidney** (mean dose)

### Study Outcomes

#### Main Study Objective

To assess response to single agent ^177^Lutetium-DOTATATE treatment in patients with relapsed or refractory high-risk neuroblastoma.

#### Secondary Study Objectives

- To assess long term survival and response.
- To assess treatment-related toxicity.

#### Sub Study Objectives

- To correlate tumor dosimetry with response.
- To correlate somatostatin type 2 receptor (SSTR-2) expression with ^68^Ga-DOTATOC PET/CT uptake.
- To correlate the uptake on ^68^Ga-DOTATOC PET/CT with response to ^177^Lu-DOTATATE therapy.

### Adverse Events

An adverse event (AE) is any untoward medical occurrence or experience in a patient administered with an investigational product and which does not necessarily have a causal relationship with the treatment. An AE can therefore be any unfavorable and unintended sign (such as a rash or an abnormal laboratory finding) symptom, or disease temporally associated with the use of the protocol treatment. Any AE occurring during the reporting period is to be reported in the eCRF. The Investigator should assess the seriousness and causality (relatedness) of all AEs experienced by the patient and document the assessments in the patient records. Each AE should be classified with the severity grade in accordance with the NCI Common Terminology Criteria for Adverse Events (CTCAE), version 5.0 (https://ctep.cancer.gov/protocolDevelopment/electronic_applications/docs/CTCAE_v5_Quick_Reference_8.5x11.pdf). An AE not included in the CTCAE should be graded by an Investigator and recorded on the AE Form using a scale of (1) mild, (2) moderate or (3) severe. For each sign/symptom, the highest grade observed since the last visit should be recorded. Any Serious Adverse Event (SAE) occurring during the reporting period must be reported by the site investigator. Prompt initial reporting is required within 24 h after the investigator first becoming aware of the event. On becoming aware that a patient has experienced a SAE, the Investigator (or delegate) must complete, date and sign a trial specific SAE report. The report should be faxed together with a SAE fax cover sheet to Clinical Trials Office (CTO), Center for Clinical Cancer Studies (CKC), Karolinska University Hospital.

### Data Collection

Trial data is collected and entered into the eCRF system PheedIt provided by Clinical Trials Office (CTO), Center for Clinical Cancer Studies (CKC), Karolinska University Hospital. eCRFs are required to be completed for each subject enrolled in the trial and all continuously collected data will be entered in the eCRF in a timely manner, within 1 month. All data must be entered in English. The PI is responsible for ensuring the accuracy, completeness and legibility of the data recorded in the eCRFs. Study monitors, data manager or the study statistician will review data according to the Monitoring Plan and Data Management Plan. Queries that are sent to site will require response and confirmation or correction of the data by delegated site staff. The Data Management Plan provides detailed information about data collection and data management throughout the trial.

### Data Quality and Monitoring

The source documents consist of the medical records and any additional relevant documentation for trial purpose. This documentation will contain trial information such as trial number, date of informed consent, trial assignment and the name of the trial PI.

Source data for all variables will be defined in a source data log, which will be stored in the Investigator's Site File. Specific trial information such as trial title, trial number, date of informed consent, trial treatment and that the patient adheres to the inclusion and exclusion criteria should be recorded in the medical record.

### Site Set-Up and Initiation

Prior to site activation of each participating site must have an Investigator Site File with all essential documents in place, including authority approvals, Clinical Study Site Agreements and a signed delegations log. Key members of the site research team will be required to attend either a meeting or a teleconference covering aspects of the trial aim and design, protocol procedures, Adverse Event reporting, collection and reporting of data and record keeping.

### Trial Monitoring

The trial quality will be assured by trial monitoring in accordance with ICH-GCP of the trial sites in all participating countries. The monitoring will be carried out in accordance with the risk assessment and a common Monitoring Plan prepared by the monitoring unit in Sweden. At least three monitoring visits will be performed; the site initiation visit, a visit after the first included patient and a close-out visit. Additional on-site monitoring visits or more extensive source data verification may be triggered for example by poor CRF return, poor data quality, low/high SAE reporting rates, excessive number of patient withdrawals or deviations. The data recorded in the eCRFs will be controlled for consistency with the source data/hospital records during the monitoring (source data verification). Any discrepancies of data will be documented and explained in the monitoring reports.

### Audit and Inspection

The Investigator and site staff will permit trial-related monitoring, audits, ethical review, and regulatory inspection(s) at their site, providing direct access to source data/documents.

### Statistical Analysis Plan

The sample size calculation is based on a Simon Two Stage Minimax design and the primary outcome measure of response rate assessed by the Revised International Neuroblastoma Response Criteria at 1 month after completion of therapy (42, 43). A complete response + partial response rate of 40% or more is defined as the acceptable level of response. A response of 20% or less would be considered unacceptably low. The probability of obtaining a false negative result, β (i.e., incorrectly rejecting for further study a treatment with a true response rate of ≥ 40%) is set at 20%. The probability of obtaining a false positive result, α (i.e., incorrectly accepting for further study a treatment that has a true response rate ≤ 20%) is set at 10%. Stage 1 requires 14 eligible and evaluable patients, with a minimum of 3 to be responders to proceed to stage 2. A further 10 patients will be recruited in Stage 2. A minimum of 8 responses out of 24 patients would be considered success and indicate that the treatment is active and should go onto further studies to evaluate efficacy (while taking account of toxicity). A standard Simon 2 stage has been chosen over a single stage (e.g., A'Hern) design in order to allow the trial to be stopped sooner, and to prevent unnecessary exposure of patients to radiotherapy, if insufficient responses are seen. Simon Optimal (3 responses /12 patients after first stage, 8/25 at end) and Minimax (3/14, 8/24) designs gives very similar numbers, so the decision to choose the latter is largely arbitrary (one patient fewer in total, but slightly more conservative after first stage). Sample size calculations were performed using Sample Size Tables for Clinical Trials software by Machin and Campbell. Loss to follow-up is likely to be minimal; all patients are referred from clinicians at established oncology centers with excellent close relations and regular communication or the patient's continued care is at the recruiting hospital. In addition, it is current standard practice to follow-up patients until they are 18 and then refer them to adult late effect physicians (according to long term follow-up guidelines from Children's Cancer and Leukemia Group (CCLG) and other relevant guidelines).

### Analysis

Response to treatment is assessed using the Revised International Neuroblastoma Response Criteria (INRC). Overall response is defined as complete response, partial response, minor response, stable disease, or progressive disease. Baseline assessment is carried out within 2 months prior to registration and an additional ^68^Ga-DOTATOC PET/CT is performed within 2 weeks prior to treatment. Response evaluation is performed 1 month and 4 months post treatment. Overall response integrates tumor response in the primary tumor, soft tissue and bone metastases, and bone marrow. Primary and metastatic soft tissue sites are assessed by MRI or CT, ^68^Ga-DOTATOC PET/CT and ^123^I-mIBG imaging. Target and non-target lesions are defined and scored according to the RECIST 1.1 guidelines. Assessment of bone marrow involvement is done by evaluation of bilateral aspirates and bilateral trephine biopsies from a total of four sampled sites. The analysis of the primary outcome measure is carried out on an eligible and evaluable patient basis. Patients who are registered, but for any reason did not complete treatment and/or the first response evaluation at 1 month after EOT, are deemed not evaluable, and so will be excluded from the primary analysis. Outcomes for all patients will be reported on an intention-to-treat basis. Descriptive statistics are used to summarize baseline characteristics, treatment, report harms, and response outcomes.

#### Primary Outcomes Measure

- Response by the Revised International Neuroblastoma Response Criteria and RECIST 1.1 guidelines at 1 month after completion of therapy (42, 43).

#### Secondary Outcomes Measures

- Response by the Revised International Neuroblastoma Response Criteria and RECIST 1.1 guidelines at 4 months after completion of therapy.
- Progression-free Survival (PFS), defined as the time from registration until objective tumor progression or death or to date of censoring for patients who do not experience the event during trial follow-up.
- Overall Survival (OS), defined as the time from registration into the trial until date of death (death from any cause) or to date of censoring for patients who do not experience the event during trial follow-up.
- Hematological and renal toxicity according to CTCAE 5.0.

#### Sub Study Measures

- Tumor and risk organ dosimetry (SPECT/CT), defined as the absorbed dose in Gray that the tumor sites and organs of risk receive following each administration of ^177^Lu-DOTATATE.
- Semi-quantitative analysis of the expression of SSTR-2 on immunohistochemistry in the primary tumor histology. The results will be correlated with tumor uptake on ^68^Ga-DOTATOC PET/CT.
- The uptake on ^68^Ga-DOTATOC PET/CT measured by SUV_max_ (maximum standardized uptake value). Pre-treatment SUV_max_ values will be correlated to the response to ^177^Lu-DOTATATE therapy.

### Planned Interim Analysis and Timelines

The two-stage design will ensure that the study is terminated after the first stage if there is insufficient evidence of therapeutic activity. If any unexpected severe adverse events (CTC grades 3, 4) are encountered, consideration will be given to suspending the entry of new patients pending clarification of a causal relationship, and the trial may be stopped if unacceptable toxicities are observed. An interim analysis will be performed after inclusion of the first 14 patients. The main analysis of all outcome measures is planned at 6 months after completion of treatment of the last patient. In addition, subsequent analysis of all survival outcome measures will be conducted 5 years after completion of treatment of the last patient.

### Current Trial Status

Active in Sweden and Norway, recruiting since May 2021.

## Discussion

PRRT with ^177^Lu-DOTATATE is an attractive new option with potentially less adverse effects and a significantly lesser exposure to radiation for persons participating in the care of the patient, as compared to ^131^I-mIBG -therapy (36, 37, 44). Contributing factors to these findings have not been exactly defined, but probably include a lesser extent of accumulation of ^177^Lu-DOTATATE in the bone marrow, as well as a shorter biological half-life and a smaller proportion of gamma-radiation emitted by ^177^Lu as compared to ^131^I. While pilot trials on ^177^Lu-DOTATATE at UCLH, London and RCH, Melbourne have shown promising results, the phase IIa LuDO trial recently conducted at UCLH concluded that ^177^Lu-DOTATATE in children was safe, but ineffective at the given dose and dosing schedule (39–41). In the LuDO trial the administration schedule was based on what was shown to be effective in an adult neuroendocrine tumor (NET) setting with a fixed administered activity every 2 months over a period of 6 months and to be within safety limits for renal and hematological toxicity. While no objective responses were seen by the standard criteria, interim assessment did show a reduction in the mIBG semiquantitative score in three patients, indicating some effect of treatment, even if it was not sustained. Compared to NET, neuroblastoma is a rapidly proliferating tumor and the intervals between administered activity may have allowed re-population that would have masked any objective responses. While the aim was to deliver a maximum kidney dose of 23 Gy to all patients, the median kidney dose actually delivered was only 16,5 Gy with this fixed activity schedule (41). The aim of the current LuDO-N trial is to establish whether ^177^Lu-DOTATATE can be effective as a single agent, in the treatment of relapsed or primary refractory high-risk neuroblastoma, when the dose schedule is intensified to two doses delivered 2 weeks apart and the administered activity is optimized/personalized by dosimetry. Due to an anticipated increased risk of myelotoxicity, secondary to the short recovery interval between doses, autologous hematopoietic stem cells are available as stem cell support in all patients. The intensified dosing schedule is similar to the one used successfully in previous studies on ^131^I-mIBG in relapsed or primary refractory high-risk neuroblastoma and it allows for an increased dose rate as compared to the LuDO trial, without increasing the cumulative radiation dose (22).

To our knowledge, there are currently two similar clinical trials on radiopharmaceutical therapy in neuroblastoma. One is under preparation: Safety Evaluation of Peptide Receptor Radionuclide Therapy (PRRT) With ^177^Lu-DOTA0-Tyr3-Octreotate for Refractory or Recurrent Metastatic Neuroblastoma Expressing Somatostatin Receptors (NEUROBLU 02), Institut Universitaire du Cancer Toulouse – Oncopole, Toulouse, France (ClinicalTrials.gov Identifier: NCT03966651). The NEUROBLU 02 trial uses a dose escalation design to assess the effective dose and the highest dose of ^177^Lu-DOTATATE, that can be given safely without the need for stem cell re-infusion. In order to make results from the NEUROBLU 2 and LuDO-N trials comparable, the dosimetry and response evaluation protocols of these two trials have been harmonized. Another similar multi-center trial in the USA, is already recruiting: ^67^Cu-SARTATE™ Peptide Receptor Radionuclide Therapy Administered to Pediatric Patients with High-Risk, Relapsed, Refractory Neuroblastoma (ClinicalTrials.gov Identifier: NCT04023331). The trial is sponsored by Clarity Pharmaceuticals Ltd. SARTATE, similarly to DOTATATE, binds to the SSTR-2 receptor and the cytotoxic effect of ^67^Cu is, also similarly to ^177^Lu, caused by β-radiation. Imaging and response evaluation is performed using another copper isotope ^64^Cu. (45, 46). In addition, ^131^I-mIBG has been randomized as first line treatment, in two of the treatment arms of the currently ongoing Children's Oncology Group (COG) ANBL 1531 Trial (ClinicalTrials.gov Identifier: NCT03126916), in an effort to target the metastatic disease in an early stage of the treatment. The ANBL 1531 trial uses a weight-based dosing of ^131^I-mIBG (47).

While the crossfire effect of β-emitters like ^131^I, ^177^Lu or ^67^Cu, can be beneficial for treatment of bulky disease in a refractory disease or relapse setting, there is a concern, that the radiation energy might not be sufficient to cause a cytotoxic effect in single, or small clusters of metastatic cells, containing only a limited number of target antigens (48). In this situation, most of the radiation would be dispersed to surrounding healthy tissues and subsequently, the radiation dose to the metastatic cells may be insufficient. The higher linear energy transfer (LET) and significantly shorter path length of the α-particle emission from ^213^Bi or ^225^Ac, might be preferable for effective depletion of micro-metastasis, since a lethal dose to a small number of cells, can be delivered from only 1–20 α-particle traversals of the cell nucleus (48–50). Switching from PRRT based on ^131^I, ^177^Lu or ^67^Cu to targeted α-particle therapy (TAT) would, however, include a loss of the cross-fire effect caused by β-emitters, due to the significantly shorter path length of the α-particles compared to β-radiation. This is highly relevant in the context of the macroscopic and microscopic tumor heterogeneity that has been described in neuroblastoma, carrying the subsequent risk that while some tumor cells would be effectively targeted and sterilized, others might escape completely unharmed (45, 46). One possibility for delivering targeted α-particle therapy to bone metastases is to utilize osteomimetic radionuclides, such as ^223^Ra-dichloride (Xofigo®) that incorporates into newly formed bone matrix within osteoblastic metastatic lesions (51), but this might not be a feasible approach in a growing child. Alternatively, combinations of radionuclide conjugates with different targeting molecules could be explored. While there are many targeting options for neuroblastoma, the combination of ^177^Lu-DOTATATE with the already established ^131^I-mIBG therapy, could be a viable alternative for a future follow-up trial aimed at overcoming the problem of tumor heterogeneity.

## Conclusion

Overall survival in high-risk neuroblastoma is about 50% and long-term survival in the setting of primary refractory disease or relapse is exceedingly rare. Novel therapies, such as ALK-inhibitors can offer a possibility to reach secondary remission in a selected group of patients, but for the vast majority of cases there are currently no effective treatment options. We estimate that the potential benefits of ^177^Lu-DOTATATE treatment with an intensified dosing schedule, outweigh the risks in this specific group of pediatric patients with primary refractory or relapsed high-risk neuroblastoma, in whom established options for curative treatment have been exhausted. We hope that the results from the LuDO-N trial and similar trials on radiopharmaceutical therapy for high-risk neuroblastoma, will generate valuable knowledge, leading to effective therapeutic options that can significantly improve survival in the future.

## Ethics Statement

The trial will be conducted according to the trial protocol, ICH-GCP, EU-directive (2001/20/EC) and applicable regulatory requirements and in accordance with the principles described in the latest version of the Declaration of Helsinki, available through the World Medical Association (WMA) website: (https://www.wma.net/policies-post/wma-declaration-of-helsinki-ethical-principles-for-medical-research-involving-human-subjects/). The patient information and informed consent-form will incorporate wording that complies with relevant data protection and privacy legislation. The protocol and patient material will be submitted to and approved by the National Ethics Committees prior to implementation. It is the responsibility of the National Coordinating Investigators to ensure that all subsequent amendments gain the necessary local approval. This does not affect the individual clinicians' responsibility to take immediate action if thought necessary to protect the health and interest of individual patients.

## Author Contributions

FS, PG, JN, MS, SW, NH, LH, PK, DG, MG, and JS conceptualized and participated in drafting the LuDO-N trial protocol. KG, KJ, JB, JR, MK, and MN contributed with valuable feedback on the trial protocol, as well as with drafting national authority applications in order to enable implementation of the trial in their respective countries. AB contributed to the dosimetry and response evaluation protocols and was responsible for setting up a system for central review of the imaging. FS, KG, MG, and JS drafted the manuscript. All authors participated in critical review of the manuscript prior to submission.

## Funding

The LuDO-N trial has been supported by funding from the Swedish Cancer Society, the Swedish Childhood Cancer Fund, Region Stockholm and Applied Accelerator Applications / Novartis. In addition, all investigational and non-investigational products have been provided free of charge for the LuDO-N trial by Applied Accelerator Applications / Novartis. MG was supported by the National Institute for Health Research, University College London Hospitals, Biomedical Research Center and the Radiation Research Unit at the Cancer Research UK City of London Center Award (C7893/A28990).

## Conflict of Interest

This study received funding from Applied Accelerator Applications, a Novartis company. The funder had the following involvement with the study: Advice on trial design, provision of all investigational and non-investigational products, free of charge for the LuDO-N trial and review of the final manuscript. The authors declare that the research was conducted in the absence of any commercial or financial relationships that could be construed as a potential conflict of interest.

## Publisher's Note

All claims expressed in this article are solely those of the authors and do not necessarily represent those of their affiliated organizations, or those of the publisher, the editors and the reviewers. Any product that may be evaluated in this article, or claim that may be made by its manufacturer, is not guaranteed or endorsed by the publisher.
